# Rescaling pain intensity measures for meta-analyses of analgesic medicines for low back pain appears justified: an empirical examination from randomised trials

**DOI:** 10.1186/s12874-022-01763-x

**Published:** 2022-11-04

**Authors:** Michael A. Wewege, Matthew D. Jones, Sam A. Williams, Steven J. Kamper, James H. McAuley

**Affiliations:** 1 School of Health Sciences, Faculty of Medicine and Health, University of New South Wales, High St, Kensington, NSW 2052 Australia; 2 Centre for Pain IMPACT, Neuroscience Research Australia, Barker St, Randwick, NSW 2031 Australia; 3 School of Health Sciences, University of Sydney, Missenden Rd, PO Box M179, Camperdown, NSW 2050 Australia; 4 Nepean Blue Mountains Local Health District, Nepean Hospital, Penrith, NSW 2750 Australia

**Keywords:** Meta-Analysis as Topic, Low Back Pain, Pain Measurement, Systematic Reviews as Topic

## Abstract

**Objective:**

Meta-analyses of analgesic medicines for low back pain often rescale measures of pain intensity to use mean difference (MD) instead of standardised mean difference for pooled estimates. Although this improves clinical interpretability, it is not clear whether this method is justified. Our study evaluated the justification for this method.

**Methods:**

We identified randomised clinical trials of analgesic medicines for adults with low back pain that used two scales with different ranges to measure the same construct of pain intensity. We transformed all data to a 0–100 scale, then compared between-group estimates across pairs of scales with different ranges.

**Results:**

Twelve trials were included. Overall, differences in means between pain intensity measures that were rescaled to a common 0–100 scale appeared to be small and randomly distributed. For one study that measured pain intensity on a 0–100 scale and a 0–10 scale; when rescaled to 0–100, the difference in MD between the scales was 0.8 points out of 100. For three studies that measured pain intensity on a 0–10 scale and 0–3 scale; when rescaled to 0–100, the average difference in MD between the scales was 0.2 points out of 100 (range 5.5 points lower to 2.7 points higher). For two studies that measured pain intensity on a 0–100 scale and a 0–3 scale; when rescaled to 0–100, the average difference in MD between the scales was 0.7 points out of 100 (range 6.2 points lower to 12.1 points higher). Finally, for six studies that measured pain intensity on a 0–100 scale and a 0–4 scale; when rescaled to 0–100, the average difference in MD between the scales was 0.7 points (range 5.4 points lower to 8.3 points higher).

**Conclusion:**

Rescaling pain intensity measures may be justified in meta-analyses of analgesic medicines for low back pain. Systematic reviewers may consider this method to improve clinical interpretability and enable more data to be included.

**Study registration/data availability:**

Open Science Framework (osf.io/8rq7f).

**Supplementary Information:**

The online version contains supplementary material available at 10.1186/s12874-022-01763-x.

## Introduction

Pain intensity is a core patient-reported outcome in low back pain clinical trials [1]. It can be quantified with several instruments including the 0 to 100 visual analogue scale (VAS); the 0 to 10 numerical rating scale (NRS); or verbal rating scales (VRS) that use qualitative classifiers (e.g., ‘no pain’, ‘mild pain’, ‘moderate pain’, ‘severe pain’), with or without corresponding numerical values [2–5]. When continuous outcome data is reported on the same scale, the pooled estimate in a meta-analysis of multiple clinical trials can be expressed as a mean difference (MD). However, when outcome data is presented on different scales, review authors typically synthesise the pooled estimate using the *standardised* mean difference (SMD), where the MD is divided by the standard deviation in the trial [6–8].

There are several limitations to the SMD [8]. First, it can be harder for stakeholders (patients, clinicians, policy makers) to clinically interpret; an MD of 1 point on an NRS is easier to interpret than an SMD of 0.4 [9]. Second, the SMD is standardised by within-study standard deviation values, which is influenced by the variability/heterogeneity in patient severity. Trials with more patient heterogeneity will have larger standard deviations, and therefore smaller SMD values, even when the unstandardised between-group differences across the trials are similar [8, 10]. This contributes to statistical heterogeneity in a meta-analysis. Similarly, standard deviations also differ depending on the type of outcome data reported in a trial (post-intervention values or change-from-baseline values), which will influence the SMD [10]. The Cochrane Handbook does not recommend pooling data presented in different types of outcome data when using the SMD (e.g., post-intervention values and change-from-baseline values) [10], which can reduce the amount of data included a meta-analysis (by removing trials when data is not presented homogenously) or increase the need for data manipulation (such as imputing alternate standard deviations in trials where data is not presented homogenously).

Given the limitations of the SMD, many reviews of analgesic medicines in adults with low back pain rescale (also known as convert or transform) all data to a common scale to enable calculation of MD as the pooled estimate [11–21]. These reviews primarily include pain intensity measured on a VAS or NRS, but some also incorporate scales with smaller ranges (e.g., 0 to 3 VRS). This approach has been justified because pain intensity measures are correlated [22], but this justification has not been properly evaluated. Therefore, we examined whether rescaling pain intensity produces comparable between-group differences in meta-analysis.

## Methods

We prospectively registered this study on the Open Science Framework on 19^th^ May 2021 (osf.io/8rq7f). The protocol, data, and code from the analysis can be found on the Open Science Framework (osf.io/8rq7f).

We used a dataset of randomised clinical trials that examined analgesic medicines in adults with low back pain from two ongoing network meta-analyses evaluating the comparative effectiveness of analgesic medicines currently licensed by regulatory agencies in the United States, United Kingdom, Europe, and Australia [13, 14]. The protocols for these two reviews have been published and provide further detail about the inclusion criteria and search strategies [13, 14]. Briefly, we searched electronic databases and clinical trials registers for randomised trials that included adults with acute or chronic non-specific low back pain, which compared an analgesic medicine to either another analgesic medicine, placebo, or no treatment. To be included in our current study, trials must have reported two or more self-report, single-item pain intensity measures on the same pain construct over the same time period (e.g., average pain at rest over the past 24 h at baseline). Given that the same participants are completing these different questions at the same timepoint(s) in the trial, this means that any difference between the scales is likely due to the different range of values.

Two authors (MAW and SAW) independently selected trials that used multiple self-report, single-item pain intensity measures at any timepoint, including baseline, which measured the same pain construct over the same time period (e.g., average pain at rest over 24 h). Scales used to measure pain intensity included the 0–100 VAS, 0–10 VAS, 0–10 NRS, and other ordinal scales (including the VRS) of different lengths. We excluded trials where the scales measured different pain constructs (e.g., VAS for pain at rest and NRS for pain during movement) or where pain intensity was judged by investigators/clinicians. Discrepancies between authors were resolved via discussion and, if necessary, with arbitration from a third author (MDJ).

From the trials included in this study, we extracted the number of participants, mean, and standard deviation for each group for the different measures of pain intensity. If pain intensity was not expressed on a 0 to 100 scale, we rescaled the measure to the 0 to 100 scale using published Eqs. [8]: the mean and standard deviation is divided by the range of the original scale and multiplied by the range of the new scale. For example, the mean and standard deviation values on a 0 to 10 scale were divided by 10 then multiplied by 100, and the mean and standard deviation values on a 0 to 3 scale were divided by 3 then multiplied by 100. If data were incomplete or unavailable, we contacted the corresponding authors of studies where possible (limited due to the age of some studies).

If qualitative classifiers (e.g., “no pain”, “mild pain”, “moderate pain”, “severe pain”) were used without corresponding numerical values, we assigned 0 to the “no pain” category and added one point to each subsequent category. In the example described in the previous sentence: “no pain” = 0, “mild pain” = 1, “moderate pain” = 2, and “severe pain” = 3. We then rescaled the data using the methods described in the previous paragraph. There were no qualitative classifiers for change-from-baseline data.

### Statistical analysis

We considered three pairs of scales for analysis, based on the reported data:

- Scales using a range of 0 to 10 (VAS or NRS) compared to scales using a range of 0 to 100 (VAS)
- Scales using a range of 0 to 10 (VAS or NRS) compared to scales using a range of 0 to < 10 (e.g., 0 to 3 VRS)
- Scales using a range of 0 to 100 (VAS) compared to scales using a range of 0 to < 10 (e.g., 0 to 3 VRS)

We estimated the between-group MD and 95% confidence intervals for the two pain intensity measures at each trial/comparison/timepoint using the *metafor* package in R with a random-effects meta-analysis model and restricted maximum likelihood estimator [23]. For placebo-controlled trials, this reflected the mean of the treatment group *minus* the mean of the placebo group. For comparative effectiveness trials, this reflected the mean of group A *minus* the mean of group B. Where trials contained more than two groups, we analysed all available combinations (e.g., A vs B, A vs C, B vs C). Within each pair of scales at each trial/comparison/timepoint, we noted the difference in the magnitude of the MD, the 95% confidence intervals, and the trial weighting. The weighting of a scale was analogous to the width of the confidence intervals. Therefore, within each pair of scales, 50% weighting would indicate no difference between the confidence intervals from each scale. We conducted a sensitivity analysis by removing trials where data had been converted (e.g., from median and interquartile range).

### Patient and public involvement

There was no patient or public involvement in this study because it was a secondary analysis of published data from clinical trials.

## Results

**Fig. 1:**
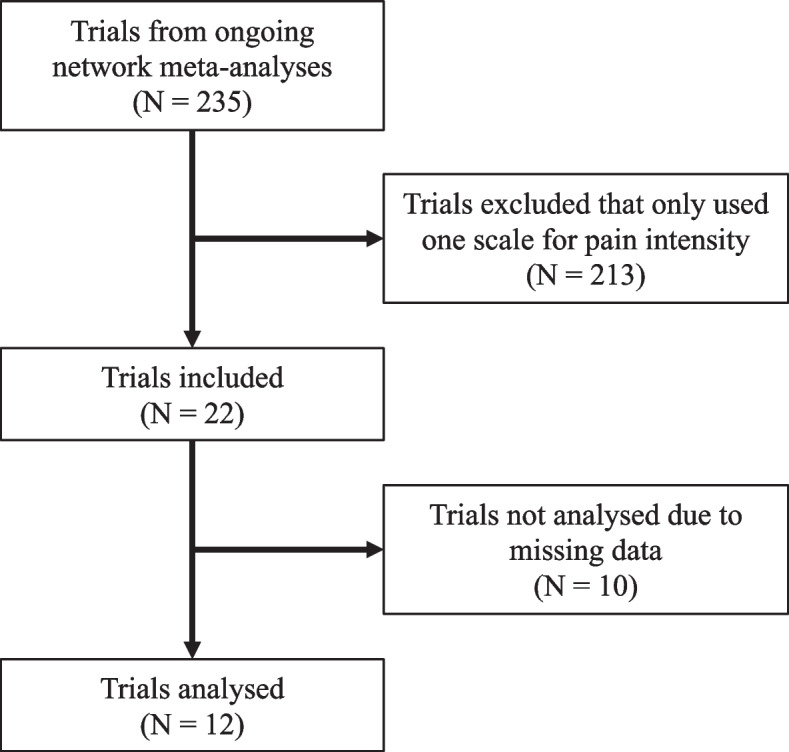
Flow diagram illustrating the literature search

The literature search is illustrated in Fig. 1. We screened 235 trials from the network meta-analyses and excluded 213 trials that only reported one scale to measure pain intensity. We identified 22 trials for inclusion, but we were unable to obtain appropriate data for analysis from 10 trials (see Additional file 1). Therefore, we included data from 12 trials that measured pain intensity at one or more timepoints using multiple measures of pain intensity for the same pain construct [3, 24–34] (Table 1). The trials included a total of 2310 participants across 24 intervention arms; nine trials contained two arms and three trials contained three arms. Three trials used a crossover design [24, 29, 30].

**Table 1:** Characteristics of included trials

Trial	Interventions (number of participants analysed)	Timepoints	Scales	Notes
Cloutier 2013 [24]	Oxycodone/naloxone (83) Placebo (83)	4 weeks	0 to 100 VAS 5-point scale ^a^	Crossover design. Analysed like parallel design with 83 participants in each group
Dreiser 2003 [25]	Diclofenac (124) Ibuprofen (122) Placebo (123)	Baseline	0 to 100 VAS 4-point scale ^b^	
Eken 2004	Acetaminophen (46) Morphine (45) Dexketoprofen (46)	Baseline 15 min 30 min	0 to 100 VAS 4-point scale ^a^	Data converted from median (range)
Friedman 2006 [27]	Methylprednisolone (39) Placebo (43)	Baseline 1 week 3 months	0 to 10 NRS 4-point scale ^b^	Individual patient data provided
Friedman 2015 [3]	Cyclobenzaprine (103) Oxycodone/acetaminophen (104) Placebo (104)	1 week 3 months	0 to 10 NRS 4-point scale ^b^	Individual patient data provided
Goforth 2014 [28]	Eszopiclone (32) Placebo (20)	Baseline 1 week 2 weeks 4 weeks	0 to 100 VAS 5-point scale ^a^	
Gordon 2010a [29]	Buprenorphine (78) Placebo (78)	4 weeks	0 to 100 VAS 5-point scale ^a^	Crossover design. Analysed like parallel design with 78 participants in each group based on ITT analysis
Gordon 2010b [30]	Buprenorphine (79) Placebo (79)	4 weeks	0 to 100 VAS 5-point scale ^a^	Crossover design. Analysed like parallel design with 79 participants in each group based on ITT analysis
Innes 1998 [31]	Ketorolac (62) Acetaminophen (60)	Baseline	0 to 100 VAS 5-point scale ^b^	
Lasko 2012 [32]	Tramadol (141) Placebo (136)	Baseline	0 to 10 NRS 4-point scale ^a^	
O’Donnell 2009 [34]	Celecoxib (396) Tramadol (396)	6 weeks	0 to 10 NRS ^c^ 0 to 100 VAS	Data presented as change from baseline
Thurel 1991 [33]	Codeine (25) Dextropropoxyphene (25)	Baseline	0 to 100 VAS 5-point scale ^b^	

^a^ Numerical values assigned to categorical pain intensity scale by trial investigators

^b^ Numerical values not assigned in the trial

^c^ Two 0 to 10 scales were used – a NRS and the modified Brief Pain Inventory

Seventeen timepoints were available for analysis (one to four per trial). One trial recorded pain intensity using two different 0 to 10 scales and one 0 to 100 scale, three trials recorded pain intensity using a 0 to 10 scale and a 4-point scale, two trials recorded pain intensity using a 0 to 100 scale and a 4-point scale, and six trials recorded pain intensity using a 0 to 100 scale and a 5-point scale. Six trials assigned numerical values to their 4-point or 5-point scales; we assigned numerical values for the other five (Table 1). Eleven included trials reported baseline and post-intervention outcome scores, and one trial reported data as change from baseline. One trial reported 4-point data as median and interquartile range, which was converted to mean and standard deviation [26].

The three crossover trials reported data for each intervention separately, as opposed to between-group paired differences, which accounts for the within-participant correlation as each participant undertook both interventions [10]. The correlation value is used in the calculation of the 95% CI and impacts the study weighting. Using the individual patient data from two included trials of different sizes, we determined that the correlation values were similar for the 0 to 10 NRS and 0 to 3 VRS (see Additional file 1). We therefore ignored within-participant correlations necessary for meta-analysis of crossover designs and analysed this data in the same way as parallel trials, which assumes that the within-participant correlations would be the same for the different scales.

Table 2 provides a summary of the findings from the analyses.

**Table 2:** Summary of findings

Figure	Pair of scales		Number of studies	Difference between MD values from scales following rescaling	Study weighting/confidence intervals
Figure 2	0 to 10	0 to 100	1	0.8 points out of 100	0 to 10 scale contributed 10–14% more weight
Figure 3	0 to 10	0 to 3 (4-point)	3	0.2 points out of 100 (range 5.5 points lower to 2.7 points higher)	0 to 3 scales contributed an average of 5.4% less weight (range 12% less to 12% more)
Figure 4	0 to 100	0 to 3 (4-point)	2	0.7 points out of 100 (range 6.2 points lower to 12.1 points higher)	0 to 3 scales contributed an average of 30.7% less weight (range 56% less to 10% more)
Figure 5	0 to 100	0 to 4 (5-point)	6	0.7 points out of 100 (range 5.4 points lower to 8.3 points higher)	0 to 4 scales contributed an average of 17% less weight (range 50% less to 18% more)

### Scales using a range of 0 to 10 compared to scales using a range of 0 to 100

**Fig. 2:**
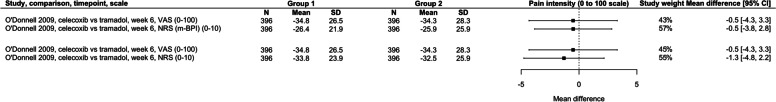
Between-group mean differences within analysis pairs from trials that used a 0–10 scale and a 0–100 scale to measure pain intensity. Study weight refers to the contribution of a scale within each analysis of study, comparison, and timepoint; 50% for each scale would suggest no difference in the precision of the scales. Scales contributing more than 50% within an analysis pair have narrower confidence intervals (more precision). CI, confidence interval; m-BPI, modified Brief Pain Inventory; N, number of participants; NRS, numerical rating scale; SD, standard deviation; VAS, visual analogue scale

One study used two 0 to 10 scales (the NRS and the modified Brief Pain Inventory “average pain”) and one 0 to 100 scale (Fig. 2). The MD from the NRS was 0.8 points lower on a 0 to 100 scale than the VAS and the study weight from the NRS was 10% greater than the VAS, reflective of smaller confidence intervals. The MD on a 0 to 100 scale was not different between the modified Brief Pain Inventory and the VAS and the study weight from the Brief Pain Inventory was 14% greater than the VAS.

### Scales using a range of 0 to 10 compared to scales using a range of 0 to < 10

**Fig. 3:**
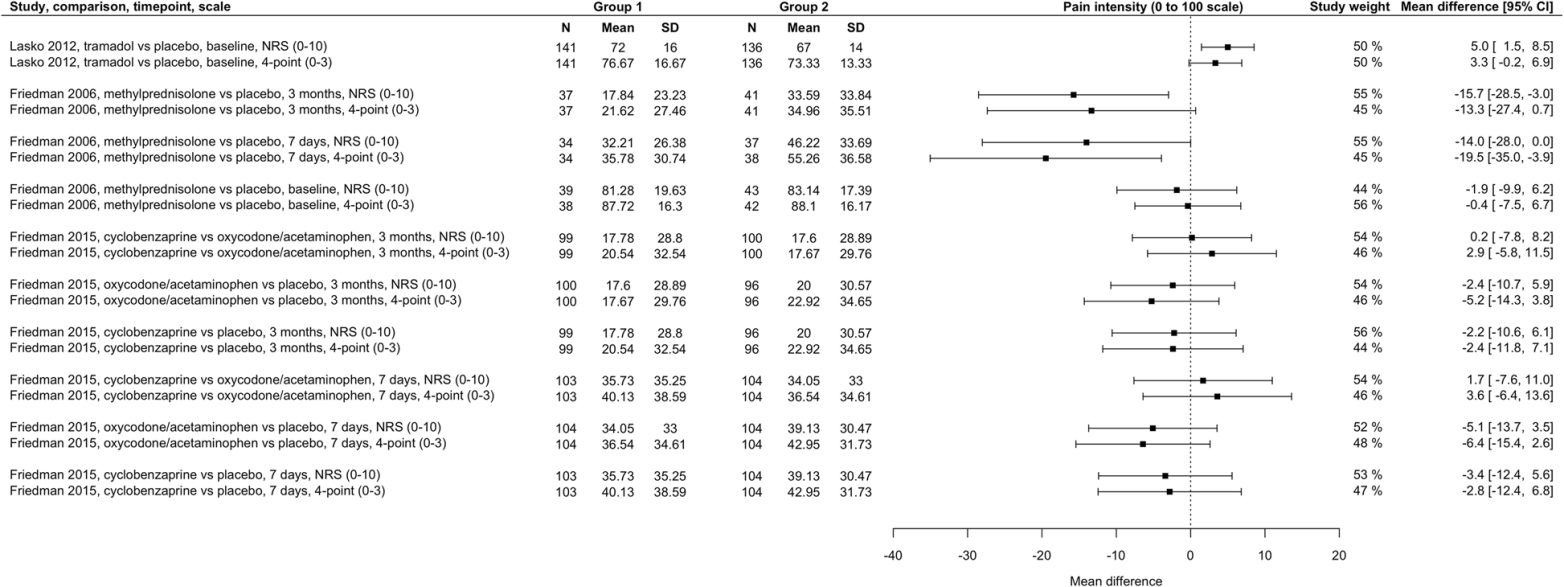
Between-group mean differences within analysis pairs from trials that used a 0–10 scale and a 4-point scale to measure pain intensity. Study weight refers to the contribution of a scale within each analysis of study, comparison, and timepoint; 50% for each scale would suggest no difference in the precision of the scales. Scales contributing more than 50% within an analysis pair have narrower confidence intervals (more precision). CI, confidence interval; N, number of participants; NRS, numerical rating scale; SD, standard deviation

Ten analysis pairs from three studies used a 0 to 10 scale and a 4-point scale (scale range 0 to 3) (Fig. 3). The average MD from 4-point scales was 0.2 points lower on a 0 to 100 scale (range 5.5 points lower to 2.7 points higher). In five pairs (50%), the MD from the 4-point scale was higher than scales using 0 to 10, and in five lower. The study weight from 4-point scales ranged from 12% smaller to 12% greater (average of differences = 5.4% smaller), reflective of wider confidence intervals. The weighting from 4-point scales was smaller than 0 to 10 scales in nine analysis pairs (80%).

### Scales using a range of 0 to 100 compared to scales using a range of 0 to < 10

Twenty-one analysis pairs from eight trials used a 0 to 100 scale and a 0 to 10 scale (two trials used 4-point scales and six trials used 5-point scales).

**Fig. 4:**
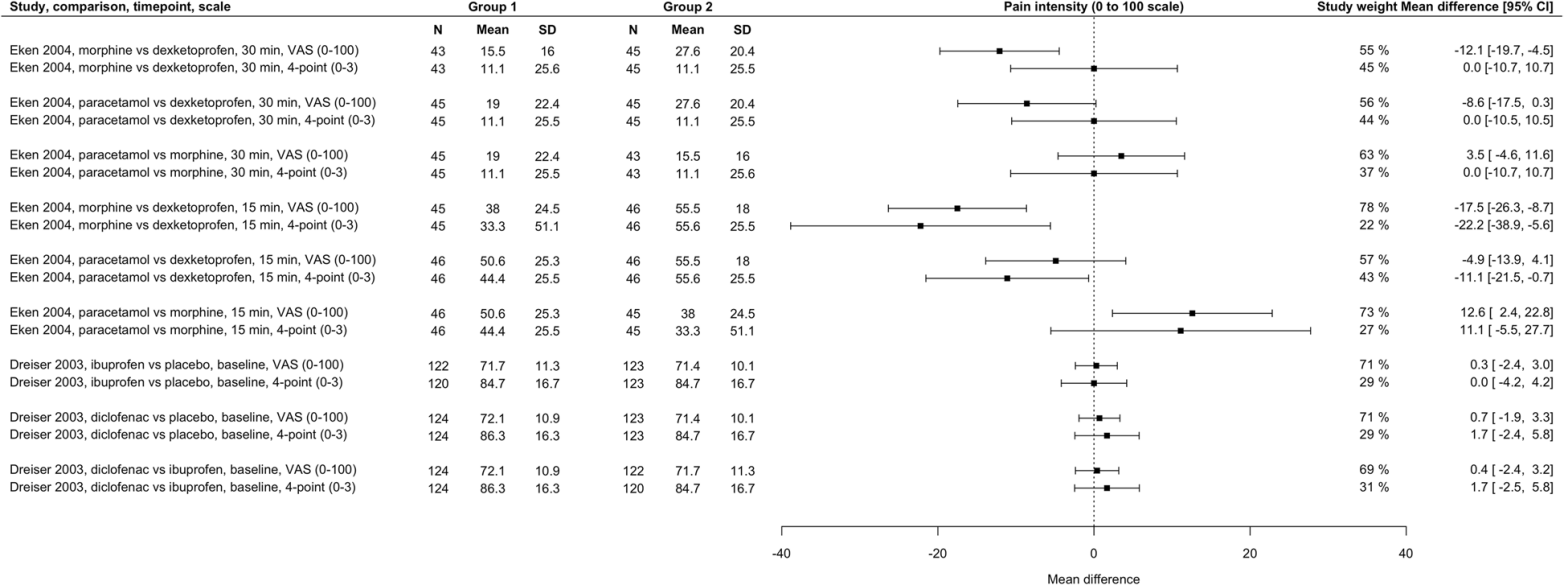
Between-group mean differences within analysis pairs from trials that used a 0–100 scale and a 4-point scale to measure pain intensity. Study weight refers to the contribution of a scale within each analysis of study, comparison, and timepoint; 50% for each scale would suggest no difference in the precision of the scales. Scales contributing more than 50% within an analysis pair have narrower confidence intervals (more precision). CI, confidence interval; N, number of participants; SD, standard deviation; VAS, visual analogue scale

Twelve analysis pairs compared a 0 to 100 scale and 4-point scale (scale range 0 to 3). Three analysis pairs from one trial could not be analysed because conversion from median and range resulted in SD of 0 for the 4-point scale. Therefore, nine analysis pairs were analysed (Fig. 4). The average MD from 4-point scales was 0.7 points higher on a 0 to 100 scale (range 6.2 points lower to 12.1 points higher). In four pairs (44%), the MD from the 4-point scale was higher than 0 to 100 scales, in five lower. The study weight from 4-point scales ranged from 56% smaller to 10% smaller (average = 30.7% smaller), reflective of wider confidence intervals. The weighting from 4-point scales was smaller than 0 to 100 scales in all analysis pairs.

In our sensitivity analysis, when we removed analysis pairs that had required scores on the 4-point scale to be converted from median and interquartile range, three analysis pairs from one trial remained (see Additional file 1). The average MD from 4-point scales ranged from 0.3 points lower to 1.27 points higher on a 0 to 100 scale, and the study weight from 4-point scales ranged from 42% smaller to 28% smaller.

**Fig. 5:**
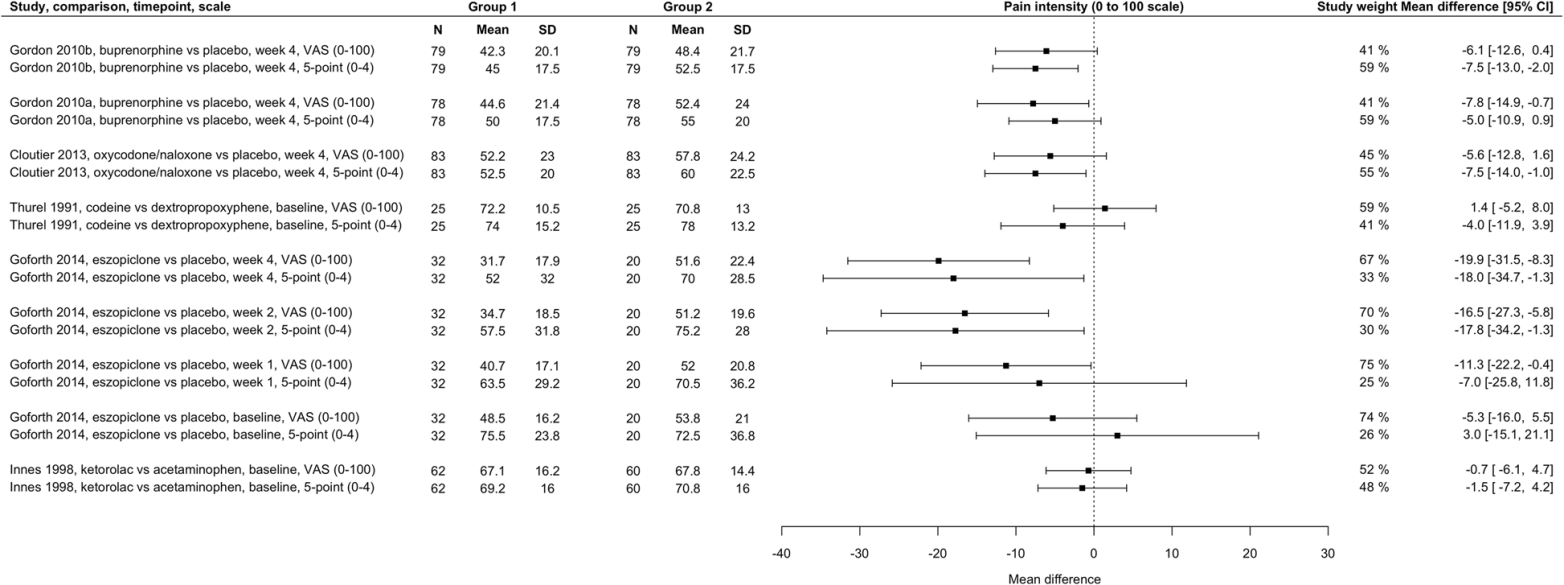
Between-group mean differences within analysis pairs from trials that used a 0–100 scale and a 5-point scale to measure pain intensity. Gordon 2010b, Gordon 2010a, and Cloutier 2013 use crossover designs; all other trials use parallel group designs. Study weight refers to the contribution of a scale within each analysis of study, comparison, and timepoint; 50% for each scale would suggest no difference in the precision of the scales. Scales contributing more than 50% within an analysis pair have narrower confidence intervals (more precision). CI, confidence interval; N, number of participants; SD, standard deviation; VAS, visual analogue scale

Nine analysis pairs compared a 0 to 100 scale and 5-point scale (scale range 0 to 4), including the three crossover trials (Fig. 5). The average MD from 5-point scales was 0.7 points higher on a 0 to 100 scale (range 5.4 points lower to 8.3 points higher). In four pairs (44%), the MD from the 5-point scale was higher than 0 to 100 scales, and in five lower. The study weight from 5-point scales ranged from 50% smaller to 18% greater (average = 17% smaller). The weighting from 5-point scales was greater than 0 to 100 scales in the three analysis pairs from crossover trials, indicating more precision from the 5-point scales.

## Discussion

The results of our study indicate rescaling pain intensity measures to a common scale may be appropriate for meta-analyses of analgesic medicines for low back pain. The method does not appear to introduce bias into the point estimate because the average differences between point estimates (the MD) across the included comparisons were small and appeared randomly distributed.

Previous reviews have primarily rescaled pain intensity for measures using ranges of 0 to 10 and 0 to 100, based on the notion that different pain intensity scales correlate well within participants [22]. The results of our study provide empirical support for this method, based on the results from one large study, and suggests that rescaling can also be extended to scales with small ranges (0 to < 10). This is important because approximately 30% of trials included in an ongoing network meta-analysis of analgesic medicines for adults with acute low back pain only used scales with smaller ranges [14]. The majority of these are older trials (published before the year 2000) but some are more recent [35–38]. The method may introduce some variability when used with smaller scales (e.g., 4-point or 5-point scales) resulting in wider confidence intervals. This may impact precision of the pooled estimate but also reduces the weight of that study; therefore, given that any additional variability would likely be balanced by other data in a meta-analysis, the impact is likely minimal.

### Implications for research

There are several benefits to using the MD in a meta-analysis of analgesic medicines for low back pain. Clinically, the results are easier to interpret by patients and clinicians [9]. Methodologically, different data formats (e.g., endpoint and change from baseline) can be readily incorporated into the meta-analysis, potentially increasing precision as well as reducing missing data and the need for data manipulation/imputation before analysis [10]. However, we believe researchers should be careful when mean and SD data from scales with small ranges (e.g., 4-point and 5-point) are not provided. We observed that converting 4-point data from median and interquartile range in one study resulted in marked discrepancies in both MD and study weighting compared to the 0 to 100 VAS, and three analyses could not be conducted due to the SD from the 4-point scale following conversion being 0. Meta-analysts should be cautious when mean and SD data from scales with small ranges (0 to < 10) are not provided. This method may also be appropriate in meta-analyses of other interventions for low back pain and other painful conditions, but further research should replicate our work in these fields. Future research could also examine the impact of this method by comparing results from rescaling to back-transforming SMD to MD, another approach that is available.

### Strengths and limitations

The strengths of this study include a prospectively registered study protocol, publicly available data/code, and comprehensive literature search underlying the evidence base of analgesic medicines for low back pain. There are several limitations. First, all included trials examined analgesic medicines in adults with low back pain, which may not generalise to other interventions or conditions (an area for future research). Second, the sample size was limited. Of the 235 trials considered for eligibility, only 22 trials were included and only 12 of these provided data necessary for analysis. Additionally, these 12 trials could be more prone to selective reporting, but a formal risk of bias assessment was not conducted in this study. Third, we made several statistical assumptions in our analysis; we assigned numerical values to categorical scales (e.g., ‘no pain’ = 0) if not reported by trial investigators. While this method closely follows how this data is reported in other trials, we assume this data can be treated as a continuous variable. Fourth, when analysing crossover trials that did not report between-group differences correctly, we assumed that the within-participants correlation could be ignored and analysed the values according to a parallel design. This has no impact on the MD, but the confidence intervals and study weight are impacted by within-participant correlation. If the correlation values are different for each scale, our results would differ. Finally, while this study only focused on single-item scales of pain intensity, function/disability scales (e.g., 0 to 100 Oswestry Disability Index, 0 to 24 Roland-Morris Disability Scale) are also frequently rescaled in meta-analysis. These constructs include much more complexity, so it is unclear whether our results hold for these scales.

## Conclusion

Our study indicates that rescaling pain intensity measures does not appear to introduce bias into the point estimate because the average differences between point estimates (the MD) across the included comparisons were small and appeared randomly distributed. The method may be appropriate for use in meta-analyses of analgesic medicines for low back pain, but further research should attempt to replicate our work in other fields.

## Supplementary Information

**Additional file 1.**

## Data Availability

The data and code used in this study are available on the Open Science Framework (osf.io/8rq7f). Additional queries can be directed to Michael Wewege (m.wewege@unsw.edu.au).

## Abbreviations

MD: Mean difference
NRS: Numerical rating scale
SD: Standard deviation
SMD: Standardised mean difference
VAS: Visual analogue scale
VRS: Verbal rating scale
