# Supplementary material for: Rescaling pain intensity measures for meta-analyses of analgesic medicines for low back pain appears justified: an empirical examination from randomised trials
Source: BMC Med Res Methodol. 2022 Nov 4;22:285. doi: 10.1186/s12874-022-01763-x (PMC9636623; doi:10.1186/s12874-022-01763-x)
Supplement: Supplementary file 1 — Additional file 1. [file 12874_2022_1763_MOESM1_ESM.docx]

**Rescaling pain intensity measures for meta-analysis appears justified: an empirical examination from randomised trials of analgesic medicines for low back pain**

**Supplementary material**

**Corresponding Author**

Michael Wewege

School of Health Sciences, Faculty of Medicine and Health

University of New South Wales

Sydney NSW Australia 2052

[m.wewege@unsw.edu.au](mailto:m.wewege@unsw.edu.au)

**Table 1.** Descriptions of eligible studies that were not included.

| **Trial** | **Scales** | **Reason for exclusion** |
| --- | --- | --- |
| Berry 1982 (1) | 0 to 10 scale, 4-pt scale | Crossover study. No author contact due to article age. |
| Berry 1988a (2) | 0 to 100 scale, 4-pt scale | Data on 4-pt scale dichotomised into groups. No author contact due to article age. |
| Berry 1988b (3) | 0 to 100 scale, 4-pt scale | Data on 4-pt scale not reported. No author contact due to article age. |
| Borenstein 1990 (4) | 0 to 20 scale, 4-pt scale | Data not presented for either scale. No author contact due to article age. |
| Castellari 1995 (5) | 0 to 100 VAS, 0 to 100 NRS, 4-pt scale | Data only presented in figures with no indication of variance. No author contact due to article age. |
| Dehghan 2015 (6) | 0 to 10 scale, VAS (range not provided) | Data not provided. No response following author contact. |
| Moore 1986 (7) | 0 to 10 scale, 8-pt scale | Data reported as sum of pain intensity differences. No author contact due to article age. |
| Ostojic 2017 (8) | 0 to 100 scale, 5-pt scale | Data on 5-pt scale dichotomised into groups. No response following author contact. |
| Stein 1996 (9) | 15-cm VAS, 7-pt scale | Data not reported for 7-pt scale. No author contact due to article age. |
| Überall 2012 (10) | 0 to 10 scale, 5-pt scale | Data on 5-pt scale not reported. No response following author contact. |

**Table 2.** Correlation values using Pearson’s product-moment correlation.

| **Study** | **Outcome** | **Results** |
| --- | --- | --- |
| Friedman 2006 (11) | Correlation between 4-pt scale at baseline and at 1 week. | *r* = -0.043 (95% CI -0.28 to 0.19), *t* = -0.36, df = 68, p = 0.72. |
|  | Correlation between 0 to 10 NRS at baseline and at 1 week. | *r* = -0.001 (95% CI -0.23 to 0.23), *t* = -0.001, df = 69, p = 0.99. |
|  | Correlation between 4-pt scale at baseline and at 1 month. | *r* = -0.06 (95% CI -0.29 to 0.16), *t* = -0.55, df = 74, p = 0.58. |
|  | Correlation between 0 to 10 NRS at baseline and at 1 month. | *r* = 0.08 (95% CI -0.14 to 0.30), *t* = 0.72, df = 76, p = 0.48. |
| Friedman 2015 (12) | Correlation between 4-pt scale at 1 week and at 3 months. | *r* = 0.30 (95% CI 0.19 to 0.40), *t* = 5.32, df = 293, p < 0.001. |
|  | Correlation between 0 to 10 NRS scale at 1 week and at 3 months. | *r* = 0.35 (95% CI 0.34 to 0.44), *t* = 6.36, df = 293, p < 0.001. |

**Figure 1.** Forest plot from the sensitivity analysis.


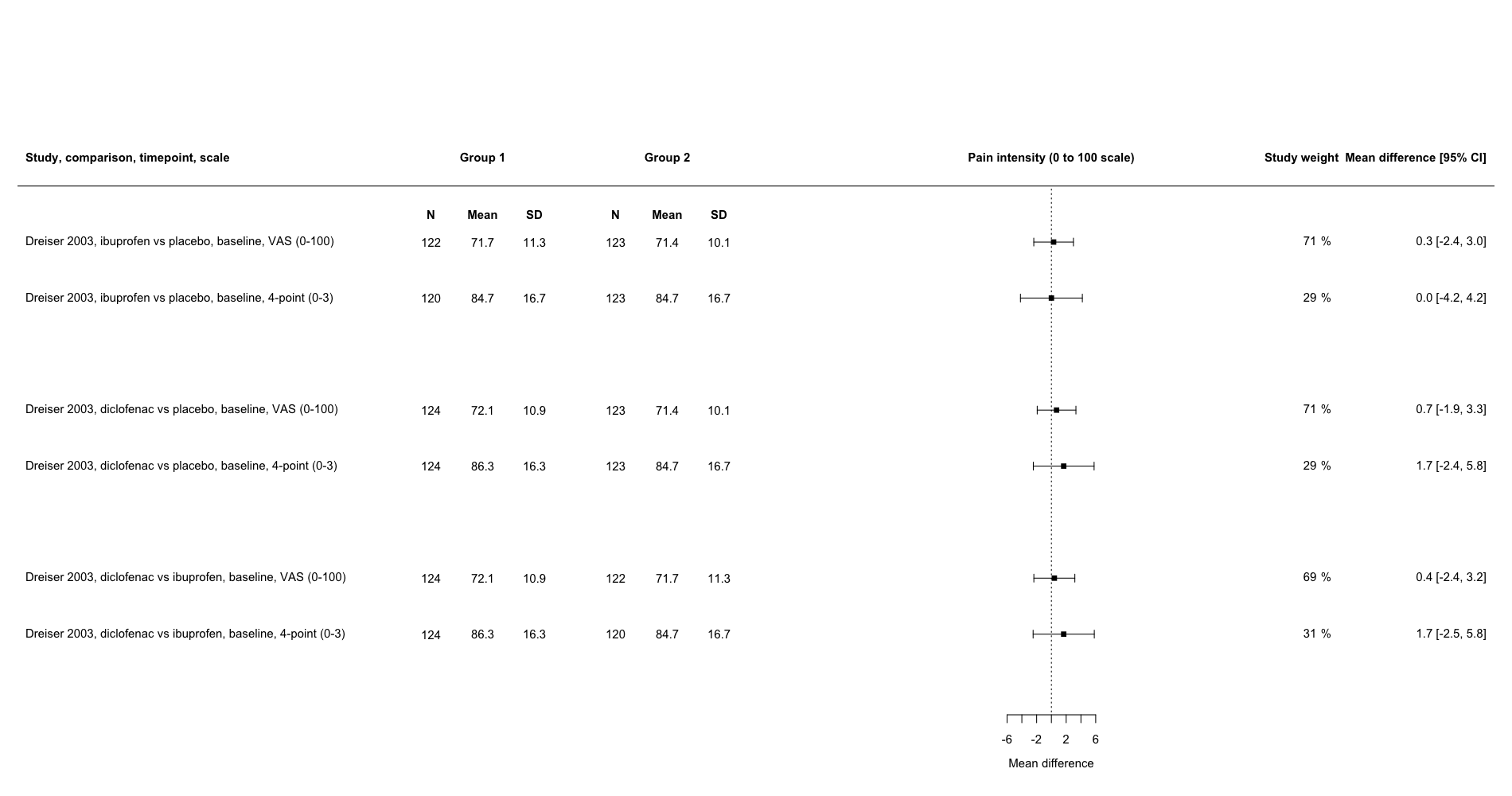


**References**

1. Berry H, Bloom B, Hamilton EB, Swinson DR. Naproxen sodium, diflunisal, and placebo in the treatment of chronic back pain. Annals of the rheumatic diseases. 1982;41(2):129-32.

2. Berry H, Hutchinson D. Tizanidine and ibuprofen in acute low-back pain: results of a double-blind multicentre study in general practice. Journal of international medical research. 1988;16(2):83-91.

3. Berry H, Hutchinson D. A multicentre placebo-controlled study in general practice to evaluate the efficacy and safety of tizanidine in acute low-back pain. Journal of international medical research. 1988;16(2):75-82.

4. Borenstein D, Lacks S, Wiesel S. Cyclobenzaprine and naproxen versus naproxen alone in the treatment of acute low back pain and muscle spasm. Clinical therapeutics. 1990;12(2):125-31.

5. Castellari A. Studio dell' efficacia antalgica di ketorolac. Uso della teletermografia nell'indagine del risultato. Giornale Italiano di Ricerche Cliniche e Terapeutiche. 1995;16(2):29-33.

6. Dehghan M, Farahbod F. Evaluation of the therapeutic effect of oral gabapentin on the severity of acute low back pain. Scientific Journal of Kurdistan University of Medical Sciences. 2015;20(1):97-104.

7. Moore RA, McQuay HJ, Carroll D, McMahon C, Allen MC. Single and Multiple Dose Analgesic and Kinetic Studies of Mefenamic Acid in Chronic Back Pain. The Clinical Journal of Pain. 1986;2(1).

8. Ostojic P, Radunovic G, Lazovic M, Tomanovic-Vujadinovic S. Ibuprofen plus paracetamol versus ibuprofen in acute low back pain: a randomized open label multicenter clinical study. Acta Reumatol Port. 2017;42(1):18-25.

9. Stein D, Peri T, Edelstein E, Elizur A, Floman Y. The efficacy of amitriptyline and acetaminophen in the management of acute low back pain. Psychosomatics. 1996;37(1):63-70.

10. Uberall MA, Mueller-Schwefe GH, Terhaag B. Efficacy and safety of flupirtine modified release for the management of moderate to severe chronic low back pain: results of SUPREME, a prospective randomized, double-blind, placebo- and active-controlled parallel-group phase IV study. Curr Med Res Opin. 2012;28(10):1617-34.

11. Friedman BW, Holden L, Esses D, Bijur PE, Choi HK, Solorzano C, et al. Parenteral corticosteroids for Emergency Department patients with non-radicular low back pain. The Journal of emergency medicine. 2006;31(4):365-70.

12. Friedman BW, Dym AA, Davitt M, Holden L, Solorzano C, Esses D, et al. Naproxen with cyclobenzaprine, oxycodone/acetaminophen, or placebo for treating acute low back pain: a randomized clinical trial. Jama. 2015;314(15):1572-80.
